# Supplementary material for: Few-shot learning for non-vitrified ice segmentation
Source: Sci Rep. 2025 Feb 14;15:5501. doi: 10.1038/s41598-025-86308-0 (PMC11828963; doi:10.1038/s41598-025-86308-0)
Supplement: Supplementary file 1 — Supplementary Information. [file 41598_2025_86308_MOESM1_ESM.pdf]

Few-Shot Learning for Non-Vitrified Ice Segmentation

Alma Vivas-Lago[1,*], Daniel Castaño-Díez[1,*]

[1]Basque Centre for Biophysics (CSIC-UPV/EHU), Bilbao, Spain.

Supplementary Information

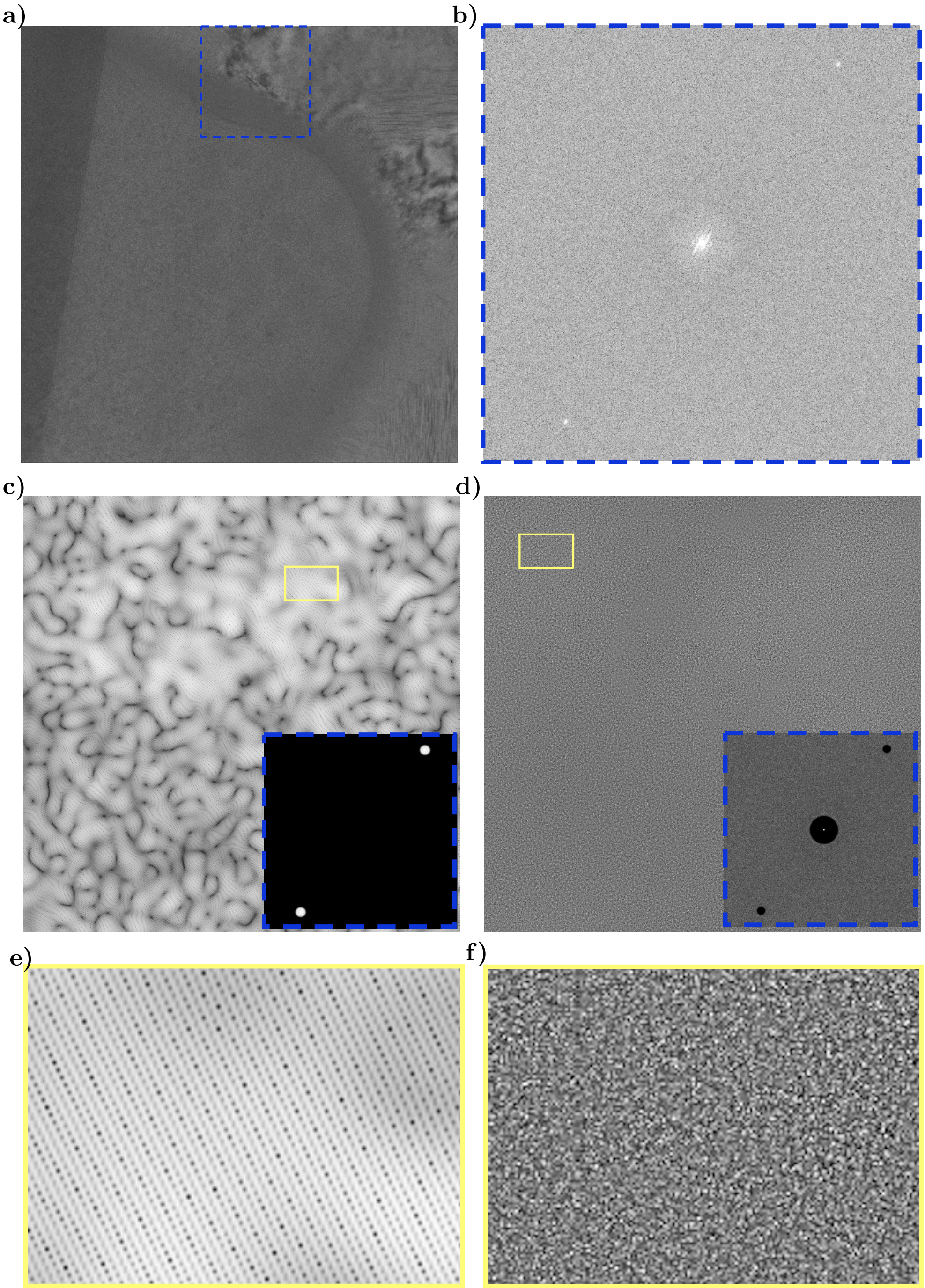

Supplementary Figure S1: TEM Characterization of Crystalline and Vitrified Ice Using Gatan's DigitalMicrograph®. a) Region of interest (blue dashed box) showing hexagonal ice (I_h), the dominant phase, with co-existing cubic (I_c) and stacking-disordered (I_{sd}) ice in other areas. b) Localized FFT of the I_h region, showing two diffraction spots and low-frequency pattern corruption. c) IFFT of diffraction peaks with the corresponding mask (shown in miniature, yellow box highlights the zoomed region in (e)). d) IFFT of vitrified ice with a mask applied to exclude diffraction peaks and low-frequency corrupted regions (shown in miniature, yellow box highlights the zoomed region in (f)). e) Zoomed-in image of (c), highlighting long-range ordered patterns characteristic of crystalline ice. f) Zoomed-in image of (d), showing the amorphous matrix characteristic of vitrified ice.

Supplementary Table S1: Examples of in situ samples in EMPIAR.

Index	EMPIAR ID	Organism/Content	Frames	Tilt Series
1	EMPIAR-11907	Saccharomyces cerevisiae	Yes	No
**2**	**EMPIAR-11830**	**Chlamydomonas reinhardtii**	**No**	**Yes**
3	EMPIAR-11756	Chlamydomonas reinhardtii	No	Yes
4	EMPIAR-11718	Red alga	No	No
5	EMPIAR-11398	Saccharomyces cerevisiae	Yes	Yes
6	EMPIAR-11658	Saccharomyces cerevisiae	No	Yes
7	EMPIAR-11198	E. amylovora	Yes	No
8	EMPIAR-11345	Mouse apoferritin	Yes	No
9	EMPIAR-10782	X-ray microCT	No	No
10	EMPIAR-10678	Yeast nucleosomes	No	Yes
11	EMPIAR-11469	Mouse islets	Yes	Yes
12	EMPIAR-11456	ChAdOx HexaPro	No	No
13	EMPIAR-11457	ChAdOx 2Pro	No	No
14	EMPIAR-11462	Yeast ER-mitochondria	Yes	Yes
15	EMPIAR-11306	HeLa cells	No	Yes
**16**	**EMPIAR-11221**	**Mouse sperm flagella**	**No**	**Yes**
17	EMPIAR-11322	Ribosome-Proteasome	No	Yes
18	EMPIAR-11324	Ribosome-Proteasome	No	Yes
19	EMPIAR-11325	E.coli	No	Yes
20	EMPIAR-11111	70S ribosomes	Yes	Yes
**21**	**EMPIAR-11166**	**S. cerevisiae autophagy**	**No**	**Yes**
22	EMPIAR-11078	C. reinhardtii ciliary	Yes	Yes
23	EMPIAR-10859	P. chlororaphis	Yes	No
24	EMPIAR-10860	E. coli APEC 2248	Yes	No
**25**	**EMPIAR-11058**	**T. kivui**	**No**	**Yes**
**26**	**EMPIAR-10987**	**80S ribosomes**	**Yes**	**Yes**
27	EMPIAR-10986	70S ribosomes	Yes	Yes
28	EMPIAR-10998	60S ribosomes	No	Yes
29	EMPIAR-10985	70S ribosomes	Yes	Yes
30	EMPIAR-10962	VPS13C	No	Yes
31	EMPIAR-10922	Mouse thalamus neurons	No	Yes
32	EMPIAR-10923	Mouse hippocampal neurons	No	Yes
33	EMPIAR-10302	Spinach tissue	No	Yes
34	EMPIAR-10377	HEK293 cells	Yes	Yes
35	EMPIAR-10378	HEK293 cells	Yes	Yes
36	EMPIAR-10713	Tissue specimen	No	Yes
37	EMPIAR-10787	Apoferritin	Yes	No
38	EMPIAR-10679	Drosophila melanogaster	No	No
39	EMPIAR-10643	HIV-1 GagdeltaMASP1T8I assemblies	Yes	Yes
40	EMPIAR-10694	C. reinhardtii	Yes	Yes
41	EMPIAR-10301	Muscle tissue (mice)	No	Yes
42	EMPIAR-10452	Rabbit skeletal muscle	Yes	Yes
43	EMPIAR-10453	Sars-Cov-2	Yes	Yes

**Index**	**EMPIAR ID**	**Organism/Content**	**Yeast**	**Tilt Series**
44	EMPIAR-10393	TMV	Yes	Yes
45	EMPIAR-10364	E. coli minicells	Yes	Yes
46	EMPIAR-10159	Yeast chromosome-segregation	Yes	Yes
47	EMPIAR-10179	Human heterochromatin	Yes	Yes
48	EMPIAR-10161	Yeast cells	Yes	Yes
49	EMPIAR-10075	Phage MS2	Yes	No

Implementation and Experimental Details

This section provides a detailed overview of the implementation specifics and experimental configurations utilized in our studies to ensure reproducibility and clarity.

Computational Setup

Our computational environment included eight NVIDIA GeForce RTX 3090 GPUs and an Intel(R) Xeon(R) Gold 6248R CPU with 96 virtual cores, supported by 500 GB of RAM.

Software and Frameworks

Experiments were conducted using Python 3.10 and PyTorch 2.0, with CUDA 11.8.

Training Specifications

Detailed configurations of the models are outlined below.

Supplementary Table S2: Training Specifications for Baseline and MAML.

**Parameter**	**Baseline**	**MAML**
Steps	6000	25,000
Epochs	N/A	7
Batch Size	12	2
Learning Rate	0.001	Outer: 9.0e-7, Inner: 5.0e-7
Scheduler	CosineAnnealingLR	None
Scheduler Parameters $T_{\max}$	10	N/A
Optimizer	SGD	Outer: Adam, Inner: SGD
Float Precision	16	16
Loss Function	SoftBCEWithLogitsLoss	SoftBCEWithLogitsLoss
Input Image Size	1024x1024	1024x1024
**Architecture**	FPN (ResNet34)	FPN (DenseNet121)
Encoder Depth	5	5
Decoder Pyramid Channels	256	256
Decoder Segmentation Channels	128	128
Decoder Dropout	0.2	0.2
Input Channels	1	1
Number of Classes	1	1
Activation Function	None	None
Upsampling Factor	4	4
Trainable Parameters	23.1M	9.3M
Estimated Model Size (MB)	92.596	37.174

Reproducibility

Reproducibility was enhanced through the use of fixed seeds and controlled environment settings:

```
pl.seed_everything(10)
torch.backends.cudnn.deterministic = True
torch.backends.cudnn.benchmark = False
```

Despite these measures, achieving 100% reproducibility remains challenging. While fixed seeds significantly reduce variability compared to random initialization, minor variations in metrics may still occur, particularly during fine-tuning. These inconsistencies arise from interactions between components implemented in pure PyTorch and those using PyTorch Lightning. For inference, however, these variations are negligible and do not affect practical outcomes.

Moreover, the quality of labels and the strategic selection of samples—especially during fine-tuning—are critical factors in achieving consistent results. This highlights the importance of careful dataset curation and thoughtful workflow design to further stabilize reproducibility and performance.

Training Durations

Training durations varied significantly across architectures and settings. DenseNet required approximately 11.3 hours under MAML conditions, while ResNet required about 6.629 hours. Baseline models trained completed in 2.45 hours for DenseNet and 1.97 hours for ResNet.
